# Developing dementia clinical pathways: considerations for health systems

**DOI:** 10.1002/alz70858_103711

**Published:** 2025-12-24

**Authors:** Kelly Kay

**Affiliations:** ^1^ Provincial Geriatrics Leadership Ontario, Toronto, ON, Canada

## Abstract

The introduction of dementia clinical pathways into healthcare systems is crucial for improving care quality, facilitating early diagnosis, and ensuring timely and appropriate interventions. The development of clinical pathways involves stakeholder engagement, current state and future needs assessment, capacity planning, clinical model and service design within a structured framework for care delivery. The identification of appropriate measures and outcomes, and a robust evaluation framework are also critical to the design of pathways that are scalable and responsive.

Co‐design with individuals living with dementia, their care partners and clinicians (e.g. neurologists, geriatricians, primary care providers, nurses, social workers etc.) can ensure that the pathway is patient‐centered, inclusive, and effective

A dementia clinical pathway cab also inform standardized diagnostic criteria and referral processes to streamline care. Clear protocols for early detection, cognitive assessments, and timely referrals to specialists can improve diagnosis and treatment. Additionally, training healthcare professionals on dementia care best practices and integrating care coordination mechanisms into electronic health records will help ensure continuity and efficiency. By identifying foundational steps and considering approaches to implementation, healthcare systems can begin the process of integrating dementia clinical pathways, ultimately improving care outcomes for individuals living with dementia.

